# Design of a Bispecific HIV Entry Inhibitor Targeting the Cell Receptor CD4 and Viral Fusion Protein Gp41

**DOI:** 10.3389/fcimb.2022.916487

**Published:** 2022-05-27

**Authors:** Hongxia Yan, Tong Wu, Yue Chen, Hongliang Jin, Li Li, Yuanmei Zhu, Huihui Chong, Yuxian He

**Affiliations:** ^1^NHC Key Laboratory of Systems Biology of Pathogens, Institute of Pathogen Biology, Chinese Academy of Medical Sciences and Peking Union Medical College, Beijing, China; ^2^Center for AIDS Research, Chinese Academy of Medical Sciences and Peking Union Medical College, Beijing, China

**Keywords:** HIV entry, bispecific inhibitor, fusion inhibitors, CD4, ibalizumab

## Abstract

Given the high variability and drug-resistance problem by human immunodeficiency virus type 1 (HIV-1), the development of bispecific or multi-specific inhibitors targeting different steps of HIV entry is highly appreciated. We previously generated a very potent short-peptide–based HIV fusion inhibitor 2P23. In this study, we designed and characterized a bifunctional inhibitor termed 2P23-iMab by genetically conjugating 2P23 to the single-chain variable fragment (scFv) of ibalizumab (iMab), a newly approved antibody drug targeting the cell receptor CD4. As anticipated, 2P23-iMab could bind to the cell membrane through CD4 anchoring and inhibit HIV-1 infection as well as viral Env-mediated cell–cell fusion efficiently. When tested against a large panel of HIV-1 pseudoviruses with different subtypes and phenotypes, 2P23-iMab exhibited dramatically improved inhibitory activity than the parental inhibitors; especially, it potently inhibited the viruses not being susceptible to iMab. Moreover, 2P23-iMab had a dramatically increased potency in inhibiting two panels of HIV-1 mutants that are resistant to T-20 or 2P23 and the infections of HIV-2 and simian immunodeficiency virus (SIV). In conclusion, our studies have provided new insights into the design of novel bispecific HIV entry inhibitors with highly potent and broad-spectrum antiviral activity.

## Introduction

There are more than 37.7 million people affected by HIV-1 globally (www.unaids.org). Despite of four decades of intense effort, an effective HIV-1 vaccine remains elusive. Combination antiretroviral therapy (cART) is effective in treating HIV-1 infection by suppressing plasma viral load below the detection limit; however, cART cannot eradicate the virus since the establishment of latent HIV-1 reservoirs, and lifelong treatment is associated with adverse effects and drug resistance (Collier et al., 2019; Deeks et al., 2021). Therefore, novel treatment strategies tend to focus on the development of antiretroviral drugs with high genetic barrier and low toxicity.

HIV-1 initiates infection with its envelope (Env) glycoproteins that are composed of surface subunit gp120 and transmembrane subunit gp41 (Xiao et al., 2021). In the process of virus entry, gp120 is responsible for binding with primary cell receptor CD4 and a coreceptor (CCR5 or CXCR4), and gp41 mediates fusion between viral and cellular membranes, leading to HIV-1 entrance into target cells. Promising data have been reported about inhibitors targeting the viral entry step (Xiao et al., 2021; Orkin et al., 2022; Xue et al., 2022). Currently, there are two small-molecule–based HIV-1 entry inhibitors clinically approved by the U.S. Food and Drug Administration, including enfuvirtide (T-20), a peptide-based fusion inhibitor targeting gp41 and maraviroc, an allosteric antagonist of CCR5. Monoclonal antibodies (MAbs) specific for virus and host proteins provide a new option with encouraging benefits in treating HIV infection, such as potent efficacy, low adverse effects, high genetic barrier for resistance, and ability to promote CD4 T-cell restoration (Sok and Burton, 2018; Tuyishime and Ferrari, 2020; Rossignol et al., 2021; Gruell and Schommers, 2022). Ibalizumab (iMab), a long-acting humanized anti-CD4 MAb, has received approval for the treatment of multidrug-resistant (MDR) HIV-infected patients when combined with other antiretroviral agents (Emu et al., 2018; Gathe et al., 2021). In a mechanism of action distinct from other drugs, including the entry inhibitors T-20 or maraviroc, iMab binds to domain 2 of CD4 and interferes with post-binding conformational changes in the gp120-CD4 complex that are required for virus-cell fusion, and thus, it blocks HIV-1 entry *via* a noncompetitive way without disturbing gp120 attachment (Moore et al., 1992). Epitope mapping revealed that two stretches of amino acids (121 to 124 and 127 to 134) in CD4 domain 2 are critical for iMab binding (Song et al., 2010). Studies on HIV-1 resistance toward iMab suggested the presence of other action mechanisms such as conformational changes of gp120 or gp120-CD4 complex (Toma et al., 2011). The crystal structure of CD4 with iMab indicated a post-coreceptor binding activity through an unknown mechanism (Freeman et al., 2010). Very importantly, the CD4 binding site of iMab is also different from that of major histocompatibility complex class II molecule interactions; thus, it does not hamper antigen presentation or CD4^+^ T cell function (Song et al., 2010; Iacob and Iacob, 2017).

HIV-1 fusion inhibitory peptides derived from the N- or C-terminal heptad repeat region (NHR or CHR) of gp41 can prevent fusion of viral and cellular membranes by blocking formation of six-helix bundle (6-HB) structure (Xiao et al., 2021). Apart from T-20, a CHR peptide-based fusion inhibitor, termed albuvirtide, has been recently approved for clinical use in China, which exhibits slightly increased anti-HIV activity over T-20 but requires infusion once weekly (Chong et al., 2012; Zhang et al., 2016; Su et al., 2020). In the past decades, our laboratory has been committed to exploiting the mechanism of HIV fusion and its inhibitors with improved pharmaceutical profiles (He et al., 2008; Chong et al., 2013; Chong et al., 2015; Chong et al., 2016; Chong et al., 2017; Ding et al., 2017; Chong et al., 2018a; Chong et al., 2018b; Zhu et al., 2018; Zhu et al., 2019; Xue et al., 2022). Among a group of newly designed fusion inhibitors, 2P23 is a CHR-derived short peptide with an M-T hook structure, and it can effectively inhibit HIV-1, HIV-2, simian immunodeficiency virus (SIV), and T-20–resistant HIV-1–mutant strains (Xiong et al., 2017). 2P23 is also a highly potent and broad-spectrum inhibitor when it is chemically or genetically modified for cell membrane anchoring or serves as a topical microbicide (Chong et al., 2017; Tang et al., 2019; Gao et al., 2021; Chen et al., 2022). Considering the high variability of HIV-1, bispecific or multi-specific antiviral inhibitors targeting different steps or epitopes of HIV-1 entry are extensively being exploited for broader coverage of the HIV-1 epidemic (Padte et al., 2018; Steinhardt et al., 2018; Tuyishime and Ferrari, 2020). Notably, iMab-based bispecific antibodies possess significantly improved antiviral activity and genetic barrier to inducing HIV-1 resistance (Pace et al., 2013b; Sun et al., 2014; Huang et al., 2016; Song et al., 2016; Moshoette et al., 2019; Li et al., 2021). In this study, we designed and characterized a bispecific HIV inhibitor by fusing the fusion inhibitor 2P23 peptide with the single-chain variable fragment (scFv) of iMab, which had dramatically increased anti-HIV activities and breadth.

## Materials and Methods

### Cells and Plasmids

HEK293T cells were purchased from the American Type Culture Collection (Rockville, MD, USA). TZM-bl cells that stably express CD4 and CCR5 along with endogenously expressed CXCR4, plasmids encoding the “global panel” HIV-1 Envs (subtypes A, B, C, G, A/C, A/E, and B/C), and molecular clones of HIV-2 (ROD and ST) were obtained through the AIDS Reagent Program, Division of AIDS, NIAID, NIH. A panel of plasmids expressing Envs derived from subtype B′ (CNE4, CNE6, CNE9, CNE11, CNE14, and CNE57), CRF01_AE (CNE107), and CRF07_BC (CNE49) was kindly provided by Linqi Zhang at the Comprehensive AIDS Research Center of Tsinghua University, Beijing, China. Four CRF07_BC Env clones (CH64.20, CH70.1, CH110, and CH120.6) were kindly provided by Yiming Shao at the Chinese Center for Disease Control and Prevention, Beijing, China. Two subtype B′ (B01 and 43-22) and one CRF01_AE (AE03) Env clones were kindly provided by Youchun Wang at the National Institute for the Control of Pharmaceutical and Biological Products, Beijing, China. Plasmids encoding two SIV Envs (mac239 and smmPBj) were kindly provided by Jianqing Xu at the Institutes of Biomedical Sciences of Fudan University, Shanghai, China. 293FT cells stably expressing CXCR4/CCR5/DSP_8–11_ and a plasmid encoding DSP_1–7_ were generous gifts from Zene Matsuda at the Institute of Medical Science of University of Tokyo, Tokyo, Japan. Cells were cultured in complete growth medium containing Dulbecco’s minimal essential medium (DMEM), 10% fetal bovine serum, penicillin-streptomycin (100 U/ml), 2 mM l-glutamine, and 1 mM sodium pyruvate and were maintained at 37°C in 5% CO_2_.

### Construction of Lentiviral Vectors Expressing iMab-Based Inhibitors

The scFv of iMab was designated iMabSC. A bispecific inhibitor targeting CD4 and gp41, designated 2P23-iMab, was constructed by linking peptide 2P23 sequence to the N terminus of iMabSC through a GGGGS linker sequence of three repeats. Both iMabSC and 2P23-iMab constructs contained a secretory signal peptide sequence of IgG3 leader at the N terminus to enhance protein secretion and a C-terminal His tag for easy detection and purification. Fusion genes encoding iMabSC or 2P23-iMabSC and green fluorescent protein (GFP) linked *via* a 2A peptide signal were synthesized (SinoGenoMax, Beijing, China) and ligated between the *Bam*HI and *Sal*I sites of a self-inactivating lentiviral transfer vector (pRRLsin.PPT.hPGK.WPRE). Recombinant lentiviruses expressing fusion genes were generated as described previously (Jin et al., 2021; Chen et al., 2022). Briefly, 1.5 × 10^7^ HEK293T cells were seeded onto P-150 cell culture dishes in 25 ml of complete DMEM medium and cultured overnight. Cells were cotransfected with 50 μg of lentiviral transfer vector encoding fusion genes, 18.75 μg of packaging plasmid delta8.9 encoding Gag/Pol/Rev, and 7.5 μg of plasmid encoding vesicular stomatitis virus G envelope by a linear polyethyleneimine (PEI) transfection reagent. Then, the culture supernatant was replaced with fresh complete DMEM plus 10% FBS at 20 h posttransfection and cultured for 24 h. Next, the supernatant was collected and centrifuged at 4,000 rpm for 15 min. After filtering by a 0.45-mm filter, the supernatant was ultracentrifuged at 25,000 rpm for 2 h. The precipitated pellets were resuspended in complete DMEM containing 10% FBS and stored in aliquots at −80°C. The titers were determined with HEK293T cells by monitoring the expression of GFP by a FACSCantoII instrument (Becton-Dickinson, Mountain View, CA, USA) and were expressed as transducing units (TU) per milliliter.

### Expression and Purification of iMabSC and 2P23-iMab

To generate HEK293T cells stably expressing recombinant protein inhibitors, a total of 1 × 10^5^ HEK293T cells were seeded onto a 24-well plate and cultured overnight. Next, 1 × 10^6^ TU of recombinant lentiviruses were added to the cells with polybrene (8 μg/ml; Sigma, St. Louis, MO, USA). After culturing for 24 h, the transduced cells were thoroughly washed and cultured in complete DMEM. The 293T cells expressing the transgenes were sorted and collected by GFP expression. After culturing 48 h, recombinant proteins were purified from cell supernatants using affinity chromatography. Following elution from a nickel affinity column, protein buffer was exchanged into phosphate-buffered saline (PBS, pH 7.4) using Amicon Ultra-4 centrifugal filter units (Millipore, Billerica, MA, USA).

### SDS-PAGE and Western Blotting

To determine the purity and molecular size of purified iMabSC and 2P23-iMab, the protein samples were loaded onto a 10% Sodium dodecylsulphate polyacrylamide gel electrophoresis (SDS-PAGE) separating gel with equal mass and the gel was stained with Coomassie brilliant blue. The recombinant inhibitors were verified by Western blotting assay. Briefly, equal amounts of the purified proteins were separated by 10% SDS-PAGE gel and transferred to a nitrocellulose membrane, which was then blocked for 1 h with a 5% (wt/vol) solution of nonfat dry milk in Tris-buffered saline–Tween 20 (TBST, pH 7.4) at room temperature. The membrane was incubated with a mouse anti-His tag antibody (Sigma) at 1:3,000 dilution or 2P23 peptide-specific MAb 5F7 (4 μg/ml) overnight at 4°C. After washing three times with TBST, the membrane was incubated with IRDye 680RD-conjugated Goat anti-Mouse immunoglobulin G (IgG) antibody (Rockland, Philadelphia, Pennsylvania, USA) at 1:20,000 dilution at room temperature for 1 h. Imaging was performed by the LI-COR Odyssey imaging system (LI-COR Biosciences, Lincoln, NE, USA).

### Flow Cytometry Assay

Binding ability of inhibitors to cell membranes was determined by flow cytometry. Briefly, a peptide or protein inhibitor was added to TZM-bl cells (1 × 10^6^) and incubated at 4°C for 1 h. After washing twice with fluorescence activated cell sorting (FACS) buffer [PBS supplemented with 0.5% bovine serum albumin and 2 mM ethylene diamine tetraacetic acid (EDTA)], cells were incubated with a mouse anti-His tag antibody (Sigma) at 1:200 dilution for 1 h at 4°C. Then, the cells were washed twice and incubated with Alexa Fluor 488 rabbit anti-mouse IgG antibody (Invitrogen, Carlsbad, CA, USA) for 1 h at 4°C. Subsequently, cells were stained with allophycocyanin–conjugated mouse anti-human CD4 antibody (BD Biosciences, Franklin Lakes, NJ, USA) for 1 h at 4°C and then resuspended by FACS buffer containing 4% formaldehyde. FACS analysis was conducted with FACSCantoII instrument.

### Single-Cycle Infection Assay

The inhibitory activity of inhibitors on a panel of HIV-1 and two SIV isolates was determined by a single-cycle infection assay as described previously (Zhu et al., 2019). In brief, pseudovirions were prepared by cotransfecting HEK293T cells with an Env-encoding plasmid and a viral backbone plasmid pSG3^Δenv^ with a linear PEI transfection reagent. Virus-containing supernatants were harvested 48 h after transfection, and 50% tissue culture infectious dose (TCID_50_) was measured in TZM-bl cells. A peptide or protein inhibitor was prepared in three-fold dilutions and then mixed with 100 TCID_50_ of viruses. After incubation for 1 h at room temperature, the mixture was added to TZM-bl cells (10^4^ per well in a 100-µl volume) and cultured for 48 h at 37°C. The cells were harvested and lysed in reporter lysis buffer, and luciferase activity was measured using luciferase assay reagents and a luminescence counter (Promega, Madison, WI, USA). The percent inhibition of pseudovirus infection and 50% inhibitory concentration (IC_50_) of an inhibitor were calculated using GraphPad Prism software (GraphPad Software Inc., San Diego, CA, USA).

### Cell–Cell Fusion Assay

The inhibitory activity of inhibitors on HIV-1 Env-mediated cell–cell fusion was measured by a dual-split protein (DSP)–based cell fusion assay as described previously (Zhu et al., 2019). In brief, a total of 1.5 × 10^4^ HEK293T cells (effector cells) were seeded on a 96-well plate and incubated at 37°C overnight. The cells were cotransfected with an Env-expressing plasmid and a DSP_1–7_–expressing plasmid and then cultured at 37°C for 24 h. 293FT cells expressing CXCR4/CCR5 and DSP_8–11_ (target cells) were resuspended in prewarmed culture medium containing EnduRen live-cell substrate (~17 μg/ml; Promega, Madison, WI, USA) and incubated for 30 min at 37°C. Next, 3 × 10^4^ of target cells were transferred to the effector cell wells with or without a tested inhibitor at graded concentrations. The cell mixture was spun down to maximize cell–cell contact and incubated for 2 h at 37°C. Luciferase activity was measured with luciferase assay reagents and IC_50_ values were calculated as described above for the pseudoviruses.

### Inhibition of Replication-Competent HIV-2 Isolates

The antiviral activity of inhibitors against two replication-competent HIV-2 isolates (ROD and ST) was measured as described previously (Zhu et al., 2019). In brief, viral stocks were generated by transfecting viral molecular clones into HEK293T cells. After transfection 48 h, virus-containing culture supernatants were harvested, and TCID_50_ was quantitated in TZM-bl cells. Similar to the above, an inhibitor was three-fold diluted, mixed with 100 TCID_50_ of viruses, and added to TZM-bl cells. After incubation for 48 h, the cells were measured for luciferase activity and IC_50_ were accordingly calculated.

### Cytotoxicity of Peptide and Protein Inhibitors

The cytotoxicity of inhibitors on TZM-bl and 293FT cells was determined by cell counting kit-8 (CCK-8) (Abbkine, Wuhan, China). Briefly, cells were seeded at a density of 1 × 10^4^ cells per well on a 96-well tissue culture plate, and 50 μl of inhibitors at different concentrations were added to the cells. After incubation at 37°C for 48 h, 20 μl of CCK-8 solution reagent was pipetted into each well and incubated 2 h at 37°C. The absorbance was measured at 450 nm with a Multiscan MK3 microplate reader (Thermo Fisher Scientific, Waltham, MA, USA), and cell viability (percentage) was calculated.

## Results

### Design and Expression of a Bispecific Inhibitor Targeting CD4 and Gp41

**Figure 1:**
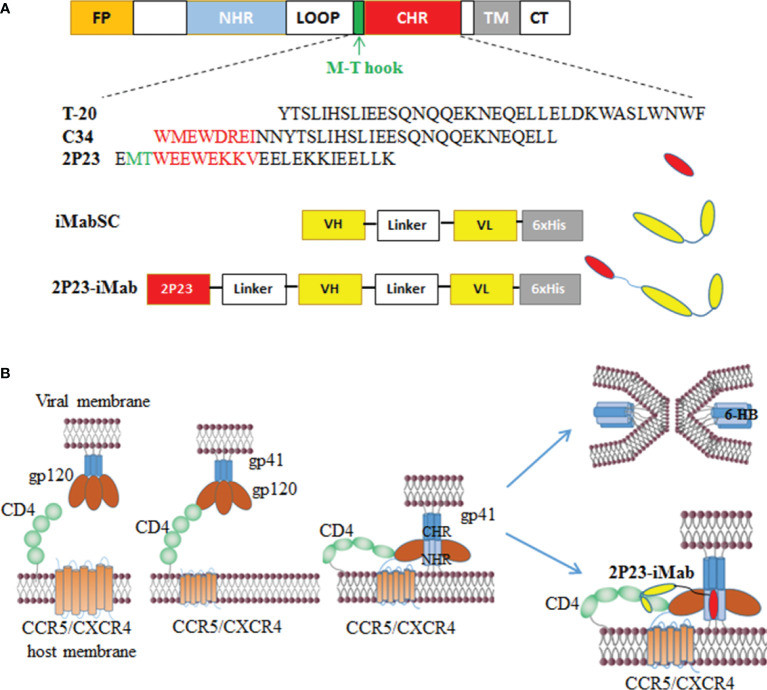
Design of bispecific HIV entry inhibitor 2P23-iMab. **(A)** Diagram of HIV-1 gp41, CHR-derived fusion inhibitor peptides (T-20, C34, 2P23), and design strategy of 2P23-iMab. FP, fusion peptide; NHR, N-terminal heptad repeat; CHR, C-terminal heptad repeat; TM, transmembrane domain. The pocket-binding sequences on C34 and 2P23 are marked in red. The position and sequence of the M-T hook structure are marked in green. iMabSC represents the single-chain variable fragment (scFv) of ibalizumab with a C-terminal His tag. 2P23-iMab represents a bispecific inhibitor by linking 2P23 to the N terminus of iMabSC. **(B)** Illustration of HIV-1 entry and action mechanism of 2P23-iMab. Binding of gp120 to the cell receptor CD4 triggers conformational changes of viral Env and further activates the fusion activity of gp41, in which FP inserts the cell membrane and CHR folds onto NHR to form a six-helix bundle (6-HB) that pulls the viral and cell membranes in close apposition for fusion. Sequentially, the binding of 2P23-iMab to CD4 makes the first strike and the binding of 2P23 to the gp41 NHR to make the second attack to the entrance of virus.

**Figure 2:**
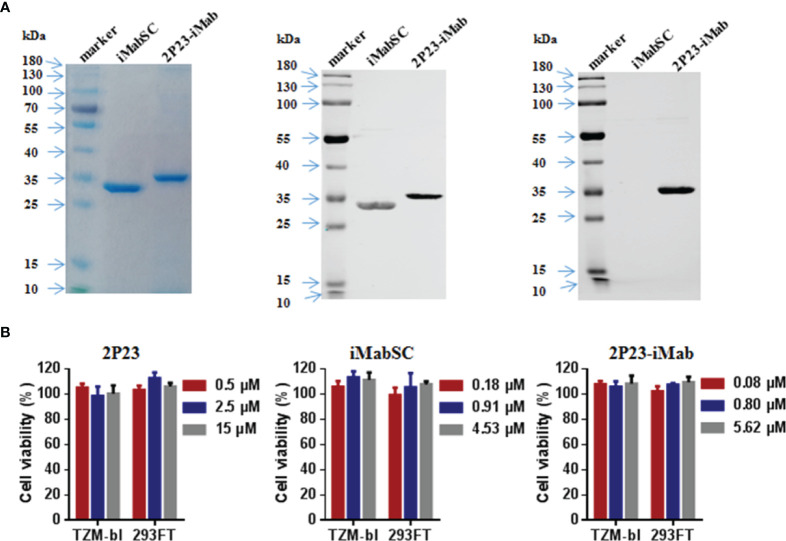
Purification and characterization of iMabSC and 2P23-iMab. **(A)** The purity and size of recombinant iMabSC and 2P23-iMab were analyzed by SDS-PAGE gel (left), and the specificity of the proteins was verified by Western blotting with a mouse anti-His antibody (middle) or anti-2P23 peptide antibody (right). **(B)** Cytotoxicity of 2P23 (left), iMabSC (middle), and 2P23-iMab (right) on TZM-bl and 293FT cell lines were determined by cell counting kit-8 (CCK-8). The experiments were performed in triplicate and repeated three times. Data are expressed as means ± standard deviations (SD).

To construct an efficient bispecific HIV entry inhibitor, the sequence encoding the fusion inhibitor 2P23 was genetically fused with sequence encoding iMabSC *via* a flexible (GGGGS)_3_ linker, thus generating a tandem fusion protein termed 2P23-iMab. Both iMabSC and 2P23-iMab proteins were designed containing a C-terminal His-tag for easy detection and purification. The schematic of two proteins is illustrated in **Figure 1A** and the mechanism of action of bispecific molecule is depicted in **Figure 1B**. As described above, HEK293T cells stably expressing iMabSC or 2P23-iMab were established with lentiviral vectors, and the recombinant proteins were purified from cell culture supernatants. The protein size and purity were visualized by 10% SDS-PAGE analysis, and the protein specificity was verified by Western blotting analysis with mouse anti-His and anti-2P23 antibodies (**Figure 2A**). Moreover, none of new protein inhibitors and synthetic 2P23 peptide showed appreciable cytotoxicity on both TZM-bl and 293FT cells at high concentrations (**Figure 2B**).

### 2P23-iMab Can Efficiently Bind to the Cell CD4 and Inhibit HIV-1 Infection

**Figure 3:**
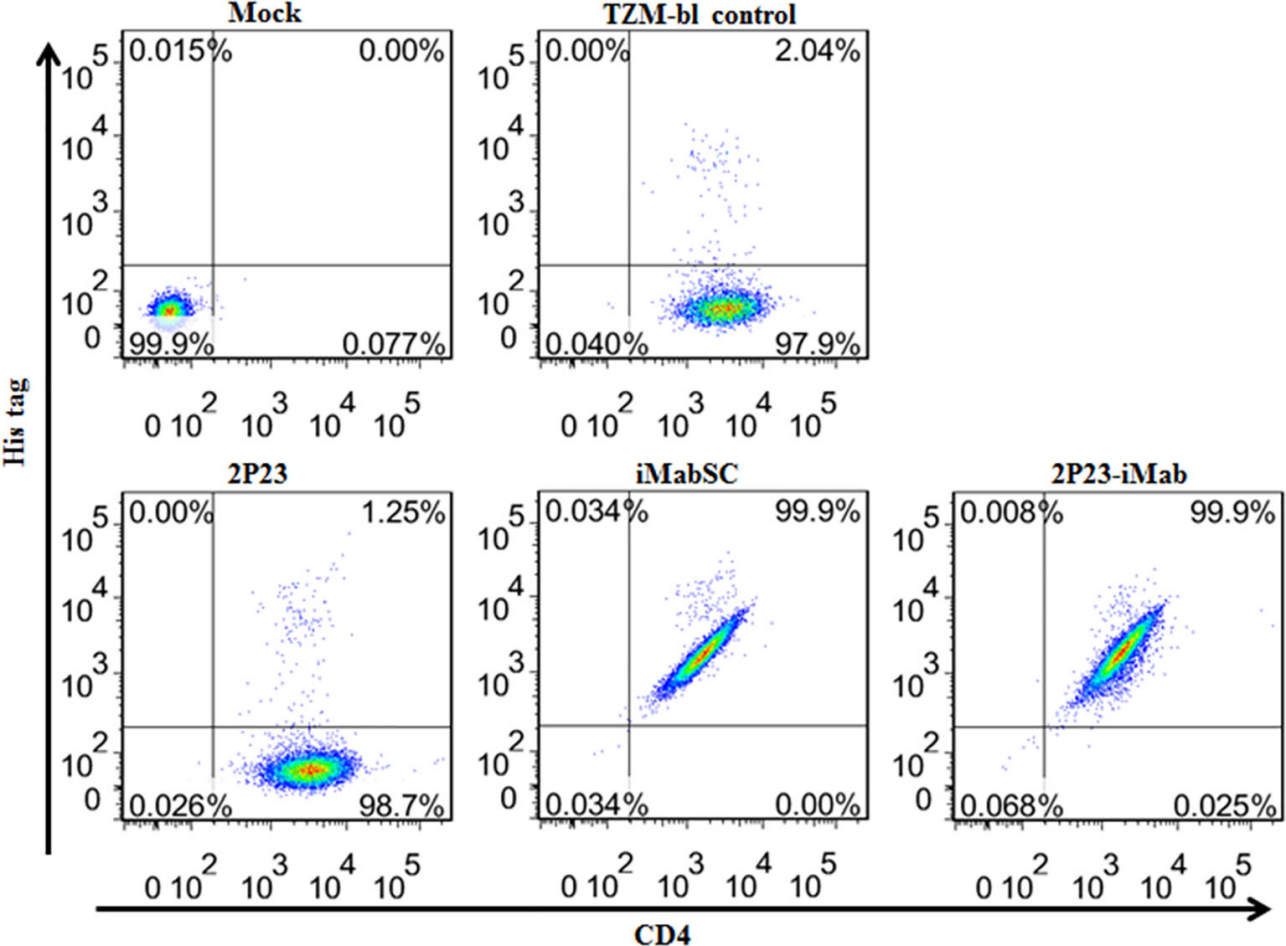
Binding abilities of iMabSC and 2P23-iMab with the target cell membrane determined by flow cytometry. TZM-bl cells were preincubated with an inhibitor for 1 h and washed thoroughly to remove unbound molecules. The binding of inhibitors was then determined by mouse anti-His tag and anti-CD4 antibodies. The fluorescence intensities of membrane-attached inhibitors were measured with a FACSCanto II instrument. Mock, TZM-bl cells only; TZM-bl control, the cells were similarly treated but an inhibitor only.

We first performed flow cytometry analysis to determine whether 2P23-iMab could bind to the target cell membrane through CD4 anchoring. To this end, an inhibitor (2P23, iMabSC, or 2P23-iMab) was pre-incubated with TZM-bl cells and then washed thoroughly to remove unbound molecules. The binding ability of the inhibitors was detected by anti-His and anti-CD4 antibodies. As shown in **Figure 3**, 2P23 did not bind to the cells significantly, whereas iMabSC and 2P23-iMab bound to the cells with the percentage of anti-His and anti-CD4 double-positive cells being 99.9%.

**Figure 4:**
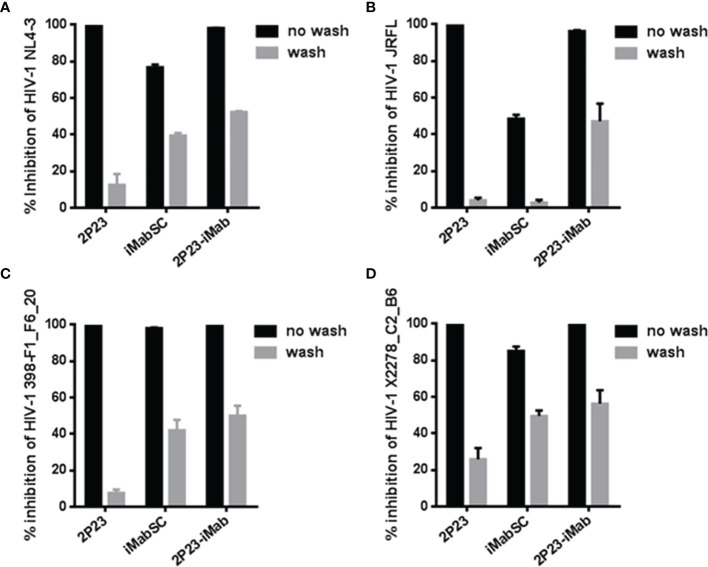
Inhibitory activities of cell membrane-anchored inhibitors against HIV-1. Similar to the described in **Figure 3**, an inhibitor was preincubated cells, followed by washes, and its sustained activity in inhibiting infection of an HIV-1 pseudovirus (NL4-3, JRFL, 398-F1_F6_20, or X2278_C2_B6) was measured by the single-cycle infection assay. The experiments were performed in triplicate and repeated three times, and data are expressed as means ± SD.

Next, we sought to determine the antiviral activity of cell-bound inhibitors by a pseudovirus-based single-cycle infection assay. A total of 1 × 10^4^ per well TZM-bl cells were plated in a 96-well plate and incubated at 37°C overnight; saturated inhibitors were added to the cells and incubated for 1 h at 37°C; then, the cells were thoroughly washed with culture medium and challenged with 100 TCID_50_ of HIV-1 pseudoviruses, including CXCR4-tropic virus NL4-3 and CCR5-tropic virus JRFL, 398-F1_F6_20, or X2278_C2_B6. As shown in **Figure 4**, if the target cells were not washed, then 2P23 and 2P23-iMab achieved complete inhibition on the infections of four viruses, whereas iMabSC could efficiently inhibit 398-F1_F6_20 but displayed less inhibitory potencies against other three viruses. When the cells were washed, 2P23, as anticipated, had a dramatically reduced anti-HIV activity, whereas the inhibitory activities of 2P23-iMab and iMabSC were largely retained except for iMabSC on JRFL infection. Taken together, these results affirmed that 2P23-iMab can efficiently bind to the cell membrane CD4 thus exerting its bifunctional anti-HIV activity.

### 2P23-iMab Is a Highly Potent Inhibitor of Diverse Primary HIV-1 Isolates

We further evaluated the antiviral activities of 2P23-iMab along with its template inhibitors by applying two large panels of primary HIV-1 pseudoviruses derived from different subtypes and phenotypes. Twelve Envs in the panel 1 (**Table 1**) were selected on the basis of the genetic and antigenic variability of viral Envs that represent the global AIDS epidemic, thus being referred to the “global panel” (deCamp et al., 2014). Twenty-one Envs in the panel 2 (**Table 2**) were derived from the subtype B and C viruses and the recombinant-forms CRF01_AE and CRF01_BC that are currently circulating in China. The corresponding Env-based pseudoviruses were prepared and a single-cycle infection assay was performed. As shown in **Table 1**, iMabSC could not effectively inhibit four viruses of the panel 1 (HIV_25710-2.43, X1632-S2-B10, CNE55, and CH119.10) as manifested as notable reductions in the maximum percent inhibition (MPI) below 50%, thus leading to a mean IC_50_ value greater than 605.28 nM. In sharp contrast, both 2P23 and 2P23-iMab potently inhibited 12 viruses with IC_50_s of 3.23 and 0.56 nM, respectively. In comparison, 2P23-iMab was about six-fold more potent than 2P23 in inhibiting divergent HIV-1 isolates. When tested against the panel 2 viruses (**Table 2**), 2P23-iMab inhibited 21 pseudoviruses with a mean IC_50_ of 0.29 nM, whereas 2P23 and iMabSC had mean IC_50_s of 3.46 and >691.24 nM, respectively. Noticeably, there were eight viruses exhibited significant reductions in susceptibility to iMabSC and manifested the MPI values below 50%. Comparing the IC_50_ values, 2P23-iMab was 12-fold more potent than 2P23 and at least 2,384-fold more potent than iMabSC. Therefore, the results verified the dramatic improved potency and breadth of the bispecific inhibitor 2P23-iMab over the parental inhibitors.

**Table 1:** Inhibitory activities of 2P23-iMab and its template inhibitors against the “global panel” HIV-1 pseudoviruses.

Pseudovirus	Subtype	Tropism	Mean IC_50_ ± SD (nM)		
			2P23	iMabSC	2P23-iMab
398-F1_F6_20	A	CCR5	1.62 ± 0.52	0.46 ± 0.11	0.32 ± 0.08
TRO.11	B	CCR5	4.35 ± 1.53	0.55 ± 0.12	0.56 ± 0.19
X2278_C2_B6	B	CCR5	0.53 ± 0.09	0.37 ± 0.05	0.11 ± 0.06
CE703010217_B6	C	CCR5	4.11 ± 1.38	0.99 ± 0.13	0.40 ± 0.16
CE1176_A3	C	CCR5	5.23 ± 0.55	0.97 ± 0.08	1.83 ± 0.88
HIV_25710-2.43	C	CCR5	2.94 ± 0.84	>1812.97	0.76 ± 0.35
X1632-S2-B10	G	CCR5	3.78 ± 0.87	>1812.97	0.26 ± 0.02
246_F3_C10_2	A/C	CCR5	4.32 ± 0.95	1.13 ± 0.08	0.76 ± 0.25
CNE8	A/E	CCR5	8.05 ± 0.96	6.32 ± 4.43	0.69 ± 0.11
CNE55	A/E	CCR5	2.34 ± 0.13	>1812.97	0.80 ± 0.20
CH119.10	B/C	CCR5	0.56 ± 0.05	>1812.97	0.07 ± 0.00
BJOX002000.03	B/C	CCR5	0.95 ± 0.13	0.64 ± 0.07	0.12 ± 0.01
Median			3.36	1.06	0.48
Mean			3.23	>605.28	0.56

The experiments were performed in triplicate and repeated three times. Data are expressed as means ± standard deviations (SD).

**Table 2:** Inhibitory activities of 2P23-iMab and its template inhibitors against divergent HIV-1 pseudoviruses.

Pseodovirus	Subtype	Tropism	Mean IC_50_ ± SD (nM)		
			2P23	iMabSC	2P23-iMab
PVO	B	CCR5	3.46 ± 0.48	0.87 ± 0.29	0.36 ± 0.07
SC422661.8	B	CCR5	1.91 ± 0.45	0.66 ± 0.34	0.12 ± 0.03
JRFL	B	CCR5	5.99 ± 1.21	>1,812.97	0.80 ± 0.24
SF162	B	CCR5	3.83 ± 0.22	>1,812.97	0.57 ± 0.07
CNE4	B'	CCR5	5.96 ± 1.14	>1,812.97	0.33 ± 0.04
CNE6	B'	CCR5	1.62 ± 0.44	2.15 ± 0.21	0.11 ± 0.05
CNE9	B'	CCR5	1.52 ± 0.40	>1812.97	0.11 ± 0.05
CNE11	B'	CCR5	7.17 ± 2.57	0.73 ± 0.11	0.78 ± 0.13
CNE14	B'	CCR5	6.60 ± 1.69	1.68 ± 0.86	0.35 ± 0.06
CNE57	B'	CCR5	2.32 ± 0.55	0.76 ± 0.22	0.14 ± 0.05
43-22	B'	CCR5	2.65 ± 0.12	0.53 ± 0.20	0.18 ± 0.02
B01	B'	CCR5	4.72 ± 0.79	0.61 ± 0.12	0.33 ± 0.11
CAP45.2.00.G3	C	CCR5	3.66 ± 0.52	0.45 ± 0.12	0.33 ± 0.20
Du156	C	CCR5	2.08 ± 0.36	0.23 ± 0.08	0.17 ± 0.02
AE03	A/E	CCR5	4.37 ± 0.77	>1,812.97	0.21 ± 0.05
CNE107	A/E	CXCR4	3.64 ± 1.56	1.21 ± 0.63	0.24 ± 0.06
CH64.20	B/C	CCR5	0.91 ± 0.22	0.82 ± 0.30	0.05 ± 0.03
CH70.1	B/C	R5/X4	3.57 ± 0.82	>1,812.97	0.15 ± 0.05
CH110	B/C	CCR5	1.28 ± 0.10	>1,812.97	0.12 ± 0.04
CH120.6	B/C	CCR5	3.93 ± 0.75	1.51 ± 0.44	0.42 ± 0.14
CNE49	B/C	CXCR4	1.53 ± 0.40	>1,812.97	0.22 ± 0.11
Median			3.57	1.51	0.22
Mean			3.46	>691.24	0.29

The experiments were performed in triplicate and repeated three times. Data are expressed as means ± SD.

### 2P23-iMab Efficiently Inhibits HIV-1 Env-Mediated Cell–Cell Fusion

To further demonstrate the advantages of 2P23-iMab, its inhibitory activity on HIV-1 Env-mediated cell–cell fusion was measured along with two templates by a DSP-based assay. Disappointedly, iMabSC could not effectively inhibit four of six Envs with a concentration as high as 1,812.97 nM, thus exhibiting a mean IC_50_ of 1,208.72 nM. In sharp contrast, 2P23 and 2P23-iMab blocked the cell–cell fusion with mean IC_50_s of 3.66 and 0.36 nM, being 330-fold or 3,358-fold more active than iMabSC. Moreover, the fusion inhibitory capacity of 2P23-iMab was about 10-fold higher than that by 2P23, which provided additional support for its improvement (**Table 3**).

**Table 3:** Inhibitory activities of 2P23-iMab and its template inhibitors against HIV-1 Env-mediated cell–cell fusion.

Env	Subtype	Tropism	Mean IC_50_ ± SD (nM)		
			2P23	iMabSC	2P23-iMab
R3A	B	R5/X4	6.42 ± 1.70	>1,812.97	0.62 ± 0.30
NL4-3	B	CXCR4	0.31 ± 0.06	>1,812.97	0.16 ± 0.04
CNE11	B'	CCR5	3.80 ± 1.17	0.16 ± 0.09	0.17 ± 0.05
CAP210.2.00.E8	C	CCR5	1.15 ± 0.42	0.30 ± 0.14	0.12 ± 0.05
CNE8	A/E	CCR5	5.75 ± 0.56	>1,812.97	0.30 ± 0.05
CH70.1	B/C	R5/X4	4.54 ± 0.97	>1,812.97	0.78 ± 0.59
Median			4.17	>1,812.97	0.24
Mean			3.66	>1,208.72	0.36

The experiments were performed in triplicate and repeated three times. Data are expressed as means ± SD.

### 2P23-iMab Is Highly Active against Drug-Resistant HIV-1 Mutants

We also evaluated 2P23-iMab for its inhibitory activity against drug-resistant HIV-1 mutants. For this aim, two panels of HIV-1 Envs bearing specific mutations that confer resistance to the fusion inhibitor T-20 or 2P23 were applied. Similarly, the corresponding pseudoviruses were prepared and used in the single-cycle infection assays. As shown, iMabSC had no appreciable inhibition on both wild type (WT) and T-20– or 2P23-resistant NL4-3 mutants, but 2P23 and 2P23-iMab were capable of inhibiting the panels of viruses efficiently (**Table 4**). In the inhibition of T-20–resistant viruses, 2P23 and 2P23-iMab showed the IC_50_s of 0.67 and 0.09 nM, respectively, which indicated a ~7-fold increased potency for 2P23-iMab over 2P23. In the inhibition of 2P23-resistant viruses, whereas 2P23 had an IC_50_ of 21 nM, 2P23-iMab had an IC_50_ of 0.55 nM, indicating a ~38-fold increased potency for 2P23-iMab. These results validated a dramatically improved activity for 2P23-iMab in inhibiting diverse fusion inhibitor-resistant HIV-1 mutants.

**Table 4:** Inhibitory activities of 2P23-iMab and its template inhibitors against HIV-1 mutants resistant to T-20 or 2P23.

NL4-3	Mean IC_50_ ± SD (nM)		
	2P23	iMabSC	2P23-iMab
**Wild-type (WT)**	0.68 ± 0.10	>1,812.97	0.09 ± 0.03
**T-20-resistant mutant**			
I37T	0.64 ± 0.09	>1,812.97	0.08 ± 0.03
V38A	0.63 ± 0.16	>1,812.97	0.06 ± 0.01
V38M	0.69 ± 0.08	>1,812.97	0.06 ± 0.02
Q40H	0.64 ± 0.09	>1,812.97	0.06 ± 0.02
N43K	0.56 ± 0.04	>1,812.97	0.10 ± 0.04
D36S/V38M	0.94 ± 0.05	>1,812.97	0.11 ± 0.04
V38A/N42T	0.42 ± 0.13	>1,812.97	0.06 ± 0.02
I37T/N43K	0.84 ± 0.10	>1,812.97	0.18 ± 0.10
**2P23-resistant mutant**			
E49A	3.14 ± 1.11	>1,812.97	0.39 ± 0.09
E49K	2.47 ± 0.06	>1,812.97	0.45 ± 0.09
Q52R	2.87 ± 0.28	>1,812.97	0.06 ± 0.02
L57R	47.39 ± 12.96	>1,812.97	1.10 ± 0.43
E136G	5.44 ± 1.12	>1,812.97	0.55 ± 0.08
N43K/E49A	2.56 ± 0.09	>1,812.97	0.37 ± 0.16
E49K/N126K	3.41 ± 1.03	>1,812.97	0.76 ± 0.05
L57R/E136G	154.51 ± 14.66	>1,812.97	0.87 ± 0.48
Q39R/N43K/N126K	1.90 ± 0.14	>1,812.97	0.51 ± 0.05
N43K/E49A/N126K	6.57 ± 0.87	>1,812.97	0.90 ± 0.57

The experiments were performed in triplicate and repeated three times. Data are expressed as means ± SD.

### 2P23-iMab Possesses Potent Activity Against HIV-2 and SIV Isolates

We were also interested in characterizing the inhibitory activity of 2P23-iMab against HIV-2 and SIV isolates. To this end, two replication-competent HIV-2 strains and two SIV pseudoviruses were applied on TZM-bl cells. As shown in **Table 5**, 2P23, iMabSC, and 2P23-iMab inhibited HIV-2_ROD_ with IC_50_s of 15.63, >1812.97, and 0.97 nM, respectively, and inhibited HIV-2_ST_ with IC_50_s of 6.94, 2.09, and 1.00 nM, respectively. The three inhibitors also blocked SIV_pbj_ with IC_50_s of 13.43, >1812.97, and 114.93 nM, respectively, and inhibited SIV239 with IC_50_s of 5.21, >1812.97, and 2.75 nM, respectively. Thus, 2P23-iMab had markedly increased activities relative to its templates in inhibiting HIV-2 and SIV infections except its inhibition on SIV_pbj_, which exhibited a ~9-fold less potency than 2P23. It is speculated that iMabSC-tethered 2P23 might suffer from a steric hindrance due to the sequence variation in the NHR of SIV_pbj_. Taken all the results together, the high ability of 2P23 to improve iMabSC was validated.

**Table 5:** Inhibitory activities of 2P23-iMab and its template inhibitors against HIV-2 and SIV isolates.

virus	Tropism	Mean IC_50_ ± SD (nM)		
		2P23	iMabSC	2P23-iMab
HIV-2_ROD_	CXCR4	15.63 ± 1.35	>1,812.97	0.97 ± 0.07
HIV-2_ST_	CCR5	6.94 ± 0.41	2.09 ± 0.33	1.00 ± 0.34
SIV_pbj_	CCR5	13.47 ± 0.97	>1,812.97	114.93 ± 8.32
SIV_239_	CCR5	5.21 ± 1.09	>1,812.97	2.75 ± 0.72

The experiments were performed in triplicate and repeated three times. Data are expressed as means ± SD.

## Discussion

In this study, we have rationally designed 2P23-iMab as a bispecific HIV entry inhibitor that can simultaneously target the primary cell receptor CD4 and fusion protein gp41. The tandem molecule consists of the short-peptide fusion inhibitor 2P23 at its N terminus and scFv of the anti-CD4 antibody iMab at the C terminus. 2P23-iMab was successfully expressed and demonstrated the integrity by SDS-PAGE and Western blotting analyses. As anticipated, 2P23-iMab could bind to the cell membrane through CD4 anchoring and inhibit HIV-1 entry efficiently. Comparing to the template inhibitors 2P23 and iMabSC, 2P23-iMab exhibited dramatically improved anti-HIV activity, as indicated by two panels of HIV-1 pseudoviruses containing 33 viral Envs derived from divergent subtypes and phenotypes (**Tables 1**, **2**). Moreover, 2P23-iMab had dramatically increased potencies in inhibiting HIV-1 Env-mediated cell–cell fusion and mutant viruses with high resistance to fusion inhibitors, as well as the infections of HIV-2 and SIV isolates. In conclusion, we think that 2P23-iMab is a bifunctional HIV entry inhibitor with highly potent and broad-spectrum antiviral activity, and thus, it possesses high potential for further development as a novel drug.

iMab, formerly known as TNX-355, was genetically engineered from its mouse progenitor mu5A8 into a human IgG4 format, which minimized the chances for CD4^+^ T-cell depletion by antibody- and complement-dependent cytotoxicity through binding to Fc receptors (Burkly et al., 1992; Boon et al., 2002). Given an equal affinity for rhesus CD4, iMab was initially evaluated in SIV-infected rhesus macaques, and it reduced plasma viremia significantly (Reimann et al., 1993; Reimann et al., 1995). In patients with HIV-1, single or multiple doses of iMab resulted in substantial reductions in viral loads and increases in CD4^+^ T-cell counts without evidence of serious adverse effects or immunologic impairments (Kuritzkes et al., 2004; Jacobson et al., 2009). A phase IIa study over 48 weeks validated the efficacy, pharmacokinetics, and safety of an iMab-based therapy in treatment-experienced adults infected with HIV-1 (Gathe et al., 2021). In a phase III study involving 40 adult patients who had advanced disease and limited treatment options with MDR HIV-1 infection, the primary end point was reached that 33 (83%) achieved a decrease in viral load of at least 0.5 log_10_ copies/ml during a 7-day treatment period, and iMab in combination with an optimized background regimen significantly reduced the viral load and increased CD4 count at week 25 (Emu et al., 2018). However, 10 out of the patients were reported with virologic failure or rebound, and the virus showed reduced susceptibility to iMab as measured by MPI (Emu et al., 2018). The previous studies reported the HIV-1 resistance toward iMab, which was associated with the loss of N-linked glycosylation sites in the V5 loop of gp120 (Fessel et al., 2011; Toma et al., 2011; Pace et al., 2013a). Interestingly, the reduced susceptibility could be restored by adding a glycan molecule in the variable region of the antibody (Song et al., 2013). Because dual targeting is thought to enhance biological efficacy, limit escape mechanisms, and increase target selectivity *via* a strong avidity effect mediated by concurrent binding, several iMab-based bispecific antibodies were created by genetically fusing iMab with broadly HIV-neutralizing antibodies (bNAbs), including anti-gp120 antibodies PG9 or PG16 (Pace et al., 2013b), m36.4 (Sun et al., 2014), CAP256 (Moshoette et al., 2019), and anti-gp41 antibody 10E8 (Huang et al., 2016), which did show greatly improved activities to inhibit HIV-1 infection and overcome the resistance problem. For the first time, here, we generated an iMab-based bifunctional inhibitor by using a fusion inhibitor peptide as a partner. The dual-targeting did render 2P23-iMab increased antiviral potency and breadth over iMab or 2P23 alone. It is conceivable that while iMab binds to CD4 to make the first strike, 2P23 targets the fusion protein gp41 to make the second attack to the entry of virus. Considering the conserved targeting site and antiviral potency by 2P23, we believe that 2P23-iMab is a highly effective inhibitor against iMab-resistant HIV-1 variants, and it might have a high genetic barrier to inducing drug-resistance either.

Herein, we would like to discuss several additional findings that are fundamentally important to the field. First, although scFv is the most common format of recombinant antibody for its preserved complete antigen-binding domains of a full-length antibody, the majority of anti-HIV bNAbs would dramatically decrease the neutralizing activity if they are produced in a scFv format (van Dorsten et al., 2020; Chen et al., 2022). Actually, it was first time to find that iMab scFv could efficiently bind the CD4 receptor and maintained or even improved the anti-HIV activity as did by a full-length IgG format (Pace et al., 2013a). Second, the previous studies showed that some Env-specific anti-HIV bNAbs exhibit the phenomenon of incomplete neutralization on particular viruses, manifesting a non-sigmoidal inhibition curve plateauing below 100% (Walker et al., 2011; Pegu et al., 2014; McCoy et al., 2015; Julg et al., 2017). Indeed, iMab and its scFv also displayed significant incomplete neutralization with the MPI values in inhibiting insensitive HIV-1 strains below 50% or more than 50% but less than 80% (Pace et al., 2013a); but encouragingly, the incomplete inhibition could be rescued by 2P23, as evidenced by the complete inhibition of 2P23-iMab against all the isolates tested in this study. Third, it is known that many bNAbs displayed sharply reduced neutralization capacity on viral Env-mediated cell–cell fusion and cell–cell HIV-1 transmission (Abela et al., 2012; Gombos et al., 2015; Reh et al., 2015), whereas fusion inhibitory peptides were highly effective inhibitors (Zhu et al., 2019; Xue et al., 2022). Our results showed that although iMabSC was not active against the majority of viral Envs to mediate cell–cell fusion, 2P23-iMab blocked all the Envs with much higher efficiency than did by 2P23 alone. Herein, it is also interesting to characterize the mechanism underlying the different susceptibility of viral Envs to the inhibition of iMabSC. Fourth, from the standpoint of a fusion inhibitor-based drug development, 2P23-iMab had the greatly increased activity than 2P23 in inhibiting divergent HIV-1 isolates, especially on T-20– and 2P23-resistant mutants. For example, 2P23-iMab was 43-fold more potent in inhibiting the L57R mutant and 178-fold more potent in inhibiting the L57R/E136G mutant. Fifth, HIV-2 has already spread to different regions worldwide and caused about 1 to 2 million infections; thus, it is very valuable to find that 2P23-iMab potently inhibited two HIV-2 isolates and two SIV isolates, whereas iMabSC inhibited one of HIV-2 only. Similarly, the mechanism underlying the ability and inability of iMabSC against HIV-2 isolates remains to be elucidated. Sixth, 2P23-iMab remains a small-size protein as compared to a full-size antibody, which would permit penetrability into tissues and lower antigenicity, and it may be more suitable for local mucosal administration.

Definitely, our present study has several limitations that need to be further addressed. First, 2P23-iMab, as a protein, might be more prone to proteases degradation, which results in short plasma half-life and poor activity. The common strategies to extend the half-life of protein drugs are fusing with human serum albumin and IgG or their derivations by taking advantage of the fact that neonatal Fc receptor (FcRn) plays a key role in albumin and IgG homeostasis. Thus, it is expected that directly conjugating 2P23 to the heavy or light chains or both of an intact-format iMab would generate a multiple-valent inhibitor with further improved anti-HIV potency and breadth. Second, toward a functional cure on HIV-1 infection, we are also working hard to develop effective gene therapy approaches with bNAbs and fusion inhibitors (Tang et al., 2019; Jin et al., 2021; Chen et al., 2022). For this aim, the *in vivo* expression of 2P23-iMab by using adeno associated virus (AAV) or lentiviral vectors will be highly appreciated. Finally, the safety profile and therapeutic efficacy of 2P23-iMab should be comprehensively evaluated in animal models before it can be advanced into clinical trials.

## Data Availability Statement

The original contributions presented in the study are included in the article/supplementary material. Further inquiries can be directed to the corresponding author.

## Author Contributions

HY performed the experiments, analyzed the data, and prepared the original draft; TW, YC, HJ, LL, YZ, and HC contributed to reagents and provided technical support. YH conceived the study and wrote the paper. All authors contributed to the article and approved the submitted version.

## Funding

This work was supported by grants from the CAMS Innovation Fund for Medical Sciences (2021-I2M-1037) and the National Natural Science Foundation of China (82002150 and 81630061).

## Conflict of Interest

The authors declare that the research was conducted in the absence of any commercial or financial relationships that could be construed as a potential conflict of interest.

## Publisher’s Note

All claims expressed in this article are solely those of the authors and do not necessarily represent those of their affiliated organizations, or those of the publisher, the editors and the reviewers. Any product that may be evaluated in this article, or claim that may be made by its manufacturer, is not guaranteed or endorsed by the publisher.

## References

[B1] Abela I. A. Berlinger L. Schanz M. Reynell L. Gunthard H. F. Rusert P.. (2012). Cell-Cell Transmission Enables HIV-1 to Evade Inhibition by Potent CD4bs Directed Antibodies. PloS Pathog. 8 (4), e1002634. doi: 10.1371/journal.ppat.1002634 22496655 PMC3320602

[B2] Boon L. Holland B. Gordon W. Liu P. Shiau F. Shanahan W.. (2002). Development of Anti-CD4 MAb Hu5a8 for Treatment of HIV-1 Infection: Preclinical Assessment in non-Human Primates. Toxicology 172 (3), 191–203. doi: 10.1016/s0300-483x(02)00002-1 11893418

[B3] Burkly L. C. Olson D. Shapiro R. Winkler G. Rosa J. J. Thomas D. W.. (1992). Inhibition of HIV Infection by a Novel CD4 Domain 2-Specific Monoclonal Antibody. Dissecting the Basis for its Inhibitory Effect on HIV-Induced Cell Fusion. J. Immunol. 149 (5), 1779–1787. 1380539

[B4] Chen Y. Jin H. Tang X. Li L. Geng X. Zhu Y.. (2022). Cell Membrane-Anchored Anti-HIV Single-Chain Antibodies and Bifunctional Inhibitors Targeting the Gp41 Fusion Protein: New Strategies for HIV Gene Therapy. Emerg. Microbes Infect. 11 (1), 30–49. doi: 10.1080/22221751.2021.2011616 34821542 PMC8735881

[B5] Chong H. Qiu Z. Su Y. Yang L. He Y. (2015). Design of a Highly Potent HIV-1 Fusion Inhibitor Targeting the Gp41 Pocket. AIDS 29 (1), 13–21. doi: 10.1097/QAD.0000000000000498 25562490

[B6] Chong H. Wu X. Su Y. He Y. (2016). Development of Potent and Long-Acting HIV-1 Fusion Inhibitors. AIDS 30 (8), 1187–1196. doi: 10.1097/QAD.0000000000001073 26919736

[B7] Chong H. Xue J. Xiong S. Cong Z. Ding X. Zhu Y.. (2017). A Lipopeptide HIV-1/2 Fusion Inhibitor With Highly Potent *In Vitro*, Ex Vivo, and *In Vivo* Antiviral Activity. J. Virol. 91 (11), e00288–17. doi: 10.1128/JVI.00288-17 28356533 PMC5432875

[B8] Chong H. Xue J. Zhu Y. Cong Z. Chen T. Guo Y.. (2018a). Design of Novel HIV-1/2 Fusion Inhibitors With High Therapeutic Efficacy in Rhesus Monkey Models. J. Virol. 92 (16), e00775–18. doi: 10.1128/JVI.00775-18 29899103 PMC6069194

[B9] Chong H. Yao X. Qiu Z. Sun J. Zhang M. Waltersperger S.. (2013). Short-Peptide Fusion Inhibitors With High Potency Against Wild-Type and Enfuvirtide-Resistant HIV-1. FASEB J. 27 (3), 1203–1213. doi: 10.1096/fj.12-222547 23233535

[B10] Chong H. Yao X. Zhang C. Cai L. Cui S. Wang Y.. (2012). Biophysical Property and Broad Anti-HIV Activity of Albuvirtide, a 3-Maleimimidopropionic Acid-Modified Peptide Fusion Inhibitor. PloS One 7 (3), e32599. doi: 10.1371/journal.pone.0032599 22403678 PMC3293837

[B11] Chong H. Zhu Y. Yu D. He Y. (2018b). Structural and Functional Characterization of Membrane Fusion Inhibitors With Extremely Potent Activity Against HIV-1, HIV-2, and Simian Immunodeficiency Virus. J. Virol. 92 (20), e01088–18. doi: 10.1128/JVI.01088-18 30089693 PMC6158425

[B12] Collier D. A. Monit C. Gupta R. K. (2019). The Impact of HIV-1 Drug Escape on the Global Treatment Landscape. Cell Host Microbe 26 (1), 48–60. doi: 10.1016/j.chom.2019.06.010 31295424

[B13] deCamp A. Hraber P. Bailer R. T. Seaman M. S. Ochsenbauer C. Kappes J.. (2014). Global Panel of HIV-1 Env Reference Strains for Standardized Assessments of Vaccine-Elicited Neutralizing Antibodies. J. Virol. 88 (5), 2489–2507. doi: 10.1128/JVI.02853-13 24352443 PMC3958090

[B14] Deeks S. G. Archin N. Cannon P. Collins S. Jones R. B. de Jong M.. (2021). Research Priorities for an HIV Cure: International AIDS Society Global Scientific Strategy 2021. Nat. Med. 27 (12), 2085–2098. doi: 10.1038/s41591-021-01590-5 34848888

[B15] Ding X. Zhang X. Chong H. Zhu Y. Wei H. Wu X.. (2017). Enfuvirtide (T20)-Based Lipopeptide Is a Potent HIV-1 Cell Fusion Inhibitor: Implication for Viral Entry and Inhibition. J. Virol. 91 (18), e00831–17. doi: 10.1128/JVI.00831-17 28659478 PMC5571253

[B16] Emu B. Fessel J. Schrader S. Kumar P. Richmond G. Win S.. (2018). Phase 3 Study of Ibalizumab for Multidrug-Resistant HIV-1. N Engl. J. Med. 379 (7), 645–654. doi: 10.1056/NEJMoa1711460 30110589

[B17] Fessel W. J. Anderson B. Follansbee S. E. Winters M. A. Lewis S. T. Weinheimer S. P.. (2011). The Efficacy of an Anti-CD4 Monoclonal Antibody for HIV-1 Treatment. Antiviral Res. 92 (3), 484–487. doi: 10.1016/j.antiviral.2011.09.010 22001594 PMC4388049

[B18] Freeman M. M. Seaman M. S. Rits-Volloch S. Hong X. Kao C. Y. Ho D. D.. (2010). Crystal Structure of HIV-1 Primary Receptor CD4 in Complex With a Potent Antiviral Antibody. Structure 18 (12), 1632–1641. doi: 10.1016/j.str.2010.09.017 21134642 PMC3005625

[B19] Gao Z. Fu R. Li X. Wang J. He Y. (2021). Safety Assessment of Microbicide 2P23 on the Rectal and Vaginal Microbiota and Its Antiviral Activity on HIV Infection. Front. Immunol. 12. doi: 10.3389/fimmu.2021.702172 PMC8382973 34447373

[B20] Gathe J. C. Hardwicke R. L. Garcia F. Weinheimer S. Lewis S. T. Cash R. B. (2021). Efficacy, Pharmacokinetics, and Safety Over 48 Weeks With Ibalizumab-Based Therapy in Treatment-Experienced Adults Infected With HIV-1: A Phase 2a Study. J. Acquir. Immune Defic. Syndr. 86 (4), 482–489. doi: 10.1097/QAI.0000000000002591 33427765 PMC7899216

[B21] Gombos R. B. Kolodkin-Gal D. Eslamizar L. Owuor J. O. Mazzola E. Gonzalez A. M.. (2015). Inhibitory Effect of Individual or Combinations of Broadly Neutralizing Antibodies and Antiviral Reagents Against Cell-Free and Cell-To-Cell HIV-1 Transmission. J. Virol. 89 (15), 7813–7828. doi: 10.1128/JVI.00783-15 25995259 PMC4505680

[B22] Gruell H. Schommers P. (2022). Broadly Neutralizing Antibodies Against HIV-1 and Concepts for Application. Curr. Opin. Virol. 54, 101211. doi: 10.1016/j.coviro.2022.101211 35306354

[B23] He Y. Cheng J. Lu H. Li J. Hu J. Qi Z.. (2008). Potent HIV Fusion Inhibitors Against Enfuvirtide-Resistant HIV-1 Strains. Proc. Natl. Acad. Sci. U S A 105 (42), 16332–16337. doi: 10.1073/pnas.0807335105 18852475 PMC2571013

[B24] Huang Y. Yu J. Lanzi A. Yao X. Andrews C. D. Tsai L.. (2016). Engineered Bispecific Antibodies With Exquisite HIV-1-Neutralizing Activity. Cell 165 (7), 1621–1631. doi: 10.1016/j.cell.2016.05.024 27315479 PMC4972332

[B25] Iacob S. A. Iacob D. G. (2017). Ibalizumab Targeting CD4 Receptors, An Emerging Molecule in HIV Therapy. Front. Microbiol. 8. doi: 10.3389/fmicb.2017.02323 PMC5711820 29230203

[B26] Jacobson J. M. Kuritzkes D. R. Godofsky E. DeJesus E. Larson J. A. Weinheimer S. P.. (2009). Safety, Pharmacokinetics, and Antiretroviral Activity of Multiple Doses of Ibalizumab (Formerly TNX-355), an Anti-CD4 Monoclonal Antibody, in Human Immunodeficiency Virus Type 1-Infected Adults. Antimicrob. Agents Chemother. 53 (2), 450–457. doi: 10.1128/AAC.00942-08 19015347 PMC2630626

[B27] Jin H. Tang X. Li L. Chen Y. Zhu Y. Chong H.. (2021). Generation of HIV-Resistant Cells With a Single-Domain Antibody: Implications for HIV-1 Gene Therapy. Cell Mol. Immunol. 18 (3), 660–674. doi: 10.1038/s41423-020-00627-y 33462383 PMC7812570

[B28] Julg B. Sok D. Schmidt S. D. Abbink P. Newman R. M. Broge T.. (2017). Protective Efficacy of Broadly Neutralizing Antibodies With Incomplete Neutralization Activity Against Simian-Human Immunodeficiency Virus in Rhesus Monkeys. J. Virol. 91 (20), e01187-17. doi: 10.1128/JVI.01187-17 28768869 PMC5625479

[B29] Kuritzkes D. R. Jacobson J. Powderly W. G. Godofsky E. DeJesus E. Haas F.. (2004). Antiretroviral Activity of the Anti-CD4 Monoclonal Antibody TNX-355 in Patients Infected With HIV Type 1. J. Infect. Dis. 189 (2), 286–291. doi: 10.1086/380802 14722894

[B30] Li S. Qiao Y. Jiang S. Wang B. Kong W. Shan Y. (2021). Broad and Potent Bispecific Neutralizing Antibody Gene Delivery Using Adeno-Associated Viral Vectors for Passive Immunization Against HIV-1. J. Control Release 338, 633–643. doi: 10.1016/j.jconrel.2021.09.006 34509584

[B31] McCoy L. E. Falkowska E. Doores K. J. Le K. Sok D. van Gils M. J.. (2015). Incomplete Neutralization and Deviation From Sigmoidal Neutralization Curves for HIV Broadly Neutralizing Monoclonal Antibodies. PloS Pathog. 11 (8), e1005110. doi: 10.1371/journal.ppat.1005110 26267277 PMC4534392

[B32] Moore J. P. Sattentau Q. J. Klasse P. J. Burkly L. C. (1992). A Monoclonal Antibody to CD4 Domain 2 Blocks Soluble CD4-Induced Conformational Changes in the Envelope Glycoproteins of Human Immunodeficiency Virus Type 1 (HIV-1) and HIV-1 Infection of CD4+ Cells. J. Virol. 66 (8), 4784–4793. doi: 10.1128/JVI.66.8.4784-4793.1992 1378510 PMC241306

[B33] Moshoette T. Ali S. A. Papathanasopoulos M. A. Killick M. A. (2019). Engineering and Characterising a Novel, Highly Potent Bispecific Antibody Imab-CAP256 That Targets HIV-1. Retrovirology 16 (1), 31. doi: 10.1186/s12977-019-0493-y 31703699 PMC6842167

[B34] Orkin C. Cahn P. Castagna A. Emu B. Harrigan P. R. Kuritzkes D. R.. (2022). Opening the Door on Entry Inhibitors in HIV: Redefining the Use of Entry Inhibitors in Heavily Treatment Experienced and Treatment-Limited Individuals Living With HIV. HIV Med. doi: 10.1111/hiv.13288 PMC9546304 35293094

[B35] Pace C. S. Fordyce M. W. Franco D. Kao C. Y. Seaman M. S. Ho D. D. (2013a). Anti-CD4 Monoclonal Antibody Ibalizumab Exhibits Breadth and Potency Against HIV-1, With Natural Resistance Mediated by the Loss of a V5 Glycan in Envelope. J. Acquir. Immune Defic. Syndr. 62 (1), 1–9. doi: 10.1097/QAI.0b013e3182732746 23023102

[B36] Pace C. S. Song R. Ochsenbauer C. Andrews C. D. Franco D. Yu J.. (2013b). Bispecific Antibodies Directed to CD4 Domain 2 and HIV Envelope Exhibit Exceptional Breadth and Picomolar Potency Against HIV-1. Proc. Natl. Acad. Sci. U S A 110 (33), 13540–13545. doi: 10.1073/pnas.1304985110 23878231 PMC3746901

[B37] Padte N. N. Yu J. Huang Y. Ho D. D. (2018). Engineering Multi-Specific Antibodies Against HIV-1. Retrovirology 15 (1), 60. doi: 10.1186/s12977-018-0439-9 30157871 PMC6114543

[B38] Pegu A. Yang Z. Y. Boyington J. C. Wu L. Ko S. Y. Schmidt S. D.. (2014). Neutralizing Antibodies to HIV-1 Envelope Protect More Effectively *In Vivo* Than Those to the CD4 Receptor. Sci. Transl. Med. 6 (243), 243ra288. doi: 10.1126/scitranslmed.3008992 PMC4562469 24990883

[B39] Reh L. Magnus C. Schanz M. Weber J. Uhr T. Rusert P.. (2015). Capacity of Broadly Neutralizing Antibodies to Inhibit HIV-1 Cell-Cell Transmission Is Strain- and Epitope-Dependent. PloS Pathog. 11 (7), e1004966. doi: 10.1371/journal.ppat.1004966 26158270 PMC4497647

[B40] Reimann K. A. Burkly L. C. Burrus B. Waite B. C. Lord C. I. Letvin N. L. (1993). *In Vivo* Administration to Rhesus Monkeys of a CD4-Specific Monoclonal Antibody Capable of Blocking AIDS Virus Replication. AIDS Res. Hum. Retroviruses 9 (3), 199–207. doi: 10.1089/aid.1993.9.199 8471310

[B41] Reimann K. A. Cate R. L. Wu Y. Palmer L. Olson D. Waite B. C.. (1995). *In Vivo* Administration of CD4-Specific Monoclonal Antibody: Effect on Provirus Load in Rhesus Monkeys Chronically Infected With the Simian Immunodeficiency Virus of Macaques. AIDS Res. Hum. Retroviruses 11 (4), 517–525. doi: 10.1089/aid.1995.11.517 7632466

[B42] Rossignol E. Alter G. Julg B. (2021). Antibodies for Human Immunodeficiency Virus-1 Cure Strategies. J. Infect. Dis. 223 (12 Suppl 2), 22–31. doi: 10.1093/infdis/jiaa165 33586772 PMC7883024

[B43] Sok D. Burton D. R. (2018). Recent Progress in Broadly Neutralizing Antibodies to HIV. Nat. Immunol. 19 (11), 1179–1188. doi: 10.1038/s41590-018-0235-7 30333615 PMC6440471

[B44] Song R. Franco D. Kao C. Y. Yu F. Huang Y. Ho D. D. (2010). Epitope Mapping of Ibalizumab, a Humanized Anti-CD4 Monoclonal Antibody With Anti-HIV-1 Activity in Infected Patients. J. Virol. 84 (14), 6935–6942. doi: 10.1128/JVI.00453-10 20463063 PMC2898252

[B45] Song R. Oren D. A. Franco D. Seaman M. S. Ho D. D. (2013). Strategic Addition of an N-Linked Glycan to a Monoclonal Antibody Improves its HIV-1-Neutralizing Activity. Nat. Biotechnol. 31 (11), 1047–1052. doi: 10.1038/nbt.2677 24097413 PMC3825789

[B46] Song R. Pace C. Seaman M. S. Fang Q. Sun M. Andrews C. D.. (2016). Distinct HIV-1 Neutralization Potency Profiles of Ibalizumab-Based Bispecific Antibodies. J. Acquir. Immune Defic. Syndr. 73 (4), 365–373. doi: 10.1097/QAI.0000000000001119 27792681 PMC5123706

[B47] Steinhardt J. J. Guenaga J. Turner H. L. McKee K. Louder M. K. O’Dell S.. (2018). Rational Design of a Trispecific Antibody Targeting the HIV-1 Env With Elevated Anti-Viral Activity. Nat. Commun. 9 (1), 877. doi: 10.1038/s41467-018-03335-4 29491415 PMC5830440

[B48] Sun M. Pace C. S. Yao X. Yu F. Padte N. N. Huang Y.. (2014). Rational Design and Characterization of the Novel, Broad and Potent Bispecific HIV-1 Neutralizing Antibody Imabm36. J. Acquir. Immune Defic. Syndr. 66 (5), 473–483. doi: 10.1097/QAI.0000000000000218 24853313 PMC4163016

[B49] Su B. Yao C. Zhao Q. X. Cai W. P. Wang M. Lu H. Z.. (2020). Efficacy and Safety of the Long-Acting Fusion Inhibitor Albuvirtide in Antiretroviral-Experienced Adults With Human Immunodeficiency Virus-1: Interim Analysis of the Randomized, Controlled, Phase 3, non-Inferiority TALENT Study. Chin. Med. J. (Engl) 133 (24), 2919–2927. doi: 10.1097/CM9.0000000000001273 33252379 PMC7752691

[B50] Tang X. Jin H. Chen Y. Li L. Zhu Y. Chong H.. (2019). A Membrane-Anchored Short-Peptide Fusion Inhibitor Fully Protects Target Cells From Infections of HIV-1, HIV-2, and Simian Immunodeficiency Virus. J. Virol. 93 (22).e01177–19. doi: 10.1128/JVI.01177-19 PMC6819927 31462566

[B51] Toma J. Weinheimer S. P. Stawiski E. Whitcomb J. M. Lewis S. T. Petropoulos C. J.. (2011). Loss of Asparagine-Linked Glycosylation Sites in Variable Region 5 of Human Immunodeficiency Virus Type 1 Envelope is Associated With Resistance to CD4 Antibody Ibalizumab. J. Virol. 85 (8), 3872–3880. doi: 10.1128/JVI.02237-10 21289125 PMC3126132

[B52] Tuyishime M. Ferrari G. (2020). Engineering Antibody-Based Molecules for HIV Treatment and Cure. Curr. Opin. HIV AIDS 15 (5), 290–299. doi: 10.1097/COH.0000000000000640 32732551 PMC7987066

[B53] van Dorsten R. T. Lambson B. E. Wibmer C. K. Weinberg M. S. Moore P. L. Morris L. (2020). Neutralization Breadth and Potency of Single-Chain Variable Fragments Derived From Broadly Neutralizing Antibodies Targeting Multiple Epitopes on the HIV-1 Envelope. J. Virol. 94 (2), e01533-19. doi: 10.1128/JVI.01533-19 31619559 PMC6955269

[B54] Walker L. M. Huber M. Doores K. J. Falkowska E. Pejchal R. Julien J. P.. (2011). Broad Neutralization Coverage of HIV by Multiple Highly Potent Antibodies. Nature 477 (7365), 466–470. doi: 10.1038/nature10373 21849977 PMC3393110

[B55] Xiao T. Cai Y. Chen B. (2021). HIV-1 Entry and Membrane Fusion Inhibitors. Viruses 13 (5), 735. doi: 10.3390/v13050735 33922579 PMC8146413

[B56] Xiong S. Borrego P. Ding X. Zhu Y. Martins A. Chong H.. (2017). A Helical Short-Peptide Fusion Inhibitor With Highly Potent Activity Against Human Immunodeficiency Virus Type 1 (HIV-1), HIV-2, and Simian Immunodeficiency Virus. J. Virol. 91 (1).e01839–16. doi: 10.1128/JVI.01839-16 27795437 PMC5165200

[B57] Xue J. Chong H. Zhu Y. Zhang J. Tong L. Lu J.. (2022). Efficient Treatment and Pre-Exposure Prophylaxis in Rhesus Macaques by an HIV Fusion-Inhibitory Lipopeptide. Cell 185(1), 131-144, e118. doi: 10.1016/j.cell.2021.11.032 34919814

[B58] Zhang H. Jin R. Yao C. Zhang T. Wang M. Xia W.. (2016). Combination of Long-Acting HIV Fusion Inhibitor Albuvirtide and LPV/r Showed Potent Efficacy in HIV-1 Patients. AIDS Res. Ther. 13, 8. doi: 10.1186/s12981-016-0091-1 26865854 PMC4748529

[B59] Zhu Y. Chong H. Yu D. Guo Y. Zhou Y. He Y. (2019). Design and Characterization of Cholesterylated Peptide HIV-1/2 Fusion Inhibitors With Extremely Potent and Long-Lasting Antiviral Activity. J. Virol. 93 (11), e02312–18. doi: 10.1128/JVI.02312-18 30867304 PMC6532087

[B60] Zhu Y. Zhang X. Ding X. Chong H. Cui S. He J.. (2018). Exceptional Potency and Structural Basis of a T1249-Derived Lipopeptide Fusion Inhibitor Against HIV-1, HIV-2, and Simian Immunodeficiency Virus. J. Biol. Chem. 293 (14), 5323–5334. doi: 10.1074/jbc.RA118.001729 29425101 PMC5892594

